# Cutting Forces Assessment in CNC Machining Processes: A Critical Review

**DOI:** 10.3390/s20164536

**Published:** 2020-08-13

**Authors:** Vitor F. C. Sousa, Francisco J. G. Silva, José S. Fecheira, Hernâni M. Lopes, Rui Pedro Martinho, Rafaela B. Casais, Luís Pinto Ferreira

**Affiliations:** ISEP–School of Engineering, Polytechnic of Porto, R. Dr. Antº Bernardino de Almeida, 431, 4200-072 Porto, Portugal; vcris@isep.ipp.pt (V.F.C.S.); jsf@isep.ipp.pt (J.S.F.); hml@isep.ipp.PT (H.M.L.); rpm@isep.ipp.pt (R.P.M.); rbc@isep.ipp.pt (R.B.C.); luispintoferreira@eu.ipp.pt (L.P.F.)

**Keywords:** CNC machining, cutting forces, cutting force prediction, finite element analysis, cutting force measurement, force sensor, dynamometer, robotic machining, process optimization

## Abstract

Machining processes remain an unavoidable technique in the production of high-precision parts. Tool behavior is of the utmost importance in machining productivity and costs. Tool performance can be assessed by the roughness left on the machined surfaces, as well as of the forces developed during the process. There are various techniques to determine these cutting forces, such as cutting force prediction or measurement, using dynamometers and other sensor systems. This technique has often been used by numerous researchers in this area. This paper aims to give a review of the different techniques and devices for measuring the forces developed for machining processes, allowing a quick perception of the advantages and limitations of each technique, through the literature research carried out, using recently published works.

## 1. Introduction

Regarding the demand for high-precision parts coming from growing industries, such as the aerospace, medical, automotive, and electrical industry [1], the CNC (Computer Numeric Control) machining market has seen considerable growth in the last six years, and it is expected to keep growing, as well as to be a $100B industry by 2025 [2]. Leading the change in the use of CNC machining processes, some countries, like India, China, and Japan, as well as some countries from the Asia Pacific area, can be pointed out [3].

This continuous growth provides an extra motivation to improve on these machining processes, from a manufacturing point of view. Knowledge about cutting forces during the machining processes has been of utmost importance to improve and optimize them, being essential when it comes to proper tool and fixture designs [4]. Cutting force values are usually obtained through empirical models that use data from machining tests, analytical models, and finite element methods (FEM). Regarding the empirical models, one of the most commonly used approaches in determining these cutting forces is by applying the Kienzle’s formula [5], being this method applied mainly in the turning process, although it can be applied to milling as well [6]. Measuring these forces can also give information on the material machinability, as well as on the optimal selection of the process parameters [7,8]. Tool behavior can also be monitored by measuring cutting forces [9]. Thus, tool optimization is also possible [10], but there are other parameters that need to be considered. Additionally, the knowledge of these cutting forces enables the control of some processing problems in the machining process, such as chatter vibration, overload, and tool condition [11]. Another advantage of monitoring these forces is that improvements can be made regarding tool geometry or even tool choice (coating choice for the correct application). Correct coating application is crucial for having a good machining process, as these coatings influence the wear rate of the tool and the quality of the machined part [12]. A wrong application or a poorly optimized process will influence the cutting forces generated during the machining process [13,14]. Having knowledge of how these forces influence the machining process is very important when it comes to process optimization, as these forces are directly correlated to certain aspects, such as surface finish quality, tool life, and energy consumption.

In this paper, a review of recently published articles is done, regarding cutting force measurement/determination during machining. The information is divided into different subchapters, each containing recent developments made in that area. Section 2 is divided into four subsections, as follows:

1. Cutting Force Prediction Methods: The prediction methods and cutting force determination models that are currently being developed are presented in this subsection;
2. Measurement of Cutting Forces: New cutting force sensors and dynamometer developments are presented in this subsection, as well as new methods of cutting force measurement and monitoring;
3. Process Optimization: It is still an important problem for the machining industry. New methods of process optimization and improvement based on the control and monitoring of cutting forces are presented;
4. Cutting Forces in Robot Machining: Cutting force assessment in robot machining is quite a novel concept, and the most recent developments made in this area are presented in this subsection.

Moreover, a summary of the research made is presented at the end of the paper, in the last section, mentioning the major advantages and drawbacks of each of the analyzed methods and identifying the common trends, observed in cutting force assessment.

## 2. Literature Review

In this section, some recent advances in cutting force determination methods regarding recently published works is presented. As previously mentioned, the main ways of determining the cutting forces are by empirical models, using analytical or numerical methods [15] and the Finite Element Method (FEM) [16]. These methods are usually employed in the prediction of cutting forces, either by analytical or mechanistic approaches, which are mentioned in detail in Section 2.1. Cutting forces can also be determined by using a force sensing system, such as a dynamometer, to monitor cutting forces in real time, during a machining process, or determine after the process, by analyzing the data collected during the cutting.

Regarding the obtention of these cutting forces, these can be derived from the current consumption measurement of the machine’s driving motors, or they can be obtained by employing various different types of dynamometers, such as piezoelectric, capacitive, optoelectrical, and strain gauge. These types of dynamometers track a certain change in a parameter due to the cutting force [17].

As mentioned in the Introduction, the main methods for cutting force determination are divided into subsections, mentioning the methods that are currently being used for cutting force prediction and cutting force modeling, as well as the methods employed for the measurement and monitoring of cutting forces. The importance of cutting force determination and prediction is well-known, being crucial for process optimization in terms of tool performance, tool design, tool life, and optimal parameter determination [4,5,7,8]. A subsection was devoted to this matter, making mention of the recent research being developed for the optimization of the cutting forces and the machining process.

Robotic machining has also seen crucial developments over recent years, and cutting force assessment in these processes is no different. There is a subsection regarding new studies made in this area, later.

### 2.1. Cutting Force Prediction Methods

Cutting force knowledge is very important when the goal is to improve/optimize a certain process, as mentioned previously. It also gives information regarding tool design and best parameters for the process, and it even enables the evaluation of the machinability of certain materials [4,5].

The prediction of cutting forces is also used to optimize certain processes, such as the machining process of gear skiving [18], or even the prediction of cutting forces in flank-milling five-axis operation [19]. Prediction of cutting forces is hard, due to the number of factors that are involved in the machining process, such as tool deflection, material, and machine configuration. An appropriate prediction of cutting forces is a key factor, especially when optimizing a machining process [20]. There is a growing need for these processes, as the complexity of some machining processes, especially five-axis machining, presents some problems, as the everchanging geometrical parameters of the cutting area. This interferes with proper data collection that some analytical models propose to solve [15]. Still regarding the prediction of cutting forces in machining, it is also important to mention that some models have been developed for the cutting force predictions when machining composites. Cutting force prediction in these materials is hard, due to their lack of homogeneity, and having the presence of isolated particulate reinforcements [21,22]. However, some mechanistic modeling approaches from metal cutting are valid when machining some composites, especially fiber-reinforced plastics [23].

Regarding FEM, this method has been employed in recent years for the prediction of cutting forces [24,25], enabling the prediction of surface roughness as well. This is very useful, as the process can be optimized in the simulation stage, not risking the waste of material/tools of making multiple tests, when using cutting force sensors. This method can be used to optimize the machining process of certain materials, giving information relative to tool temperature and chip morphology. Thus, this information can be used to optimize cutting tools. Having information on the tool temperature, coupled with the cutting force values obtained, the tool’s wear throughout the process can be analyzed. This provides the opportunity to choose the best machining parameters, such as lubrication type, to improve the tool lifespan for roughing operations or lower surface roughness values for finishing applications, regarding the optimization of cutting tools, in the turning processes. For example, different nose radii influence the cutting forces generated during machining. If these radii are smaller, then the cutting forces generated will be higher [26,27]. The study carried out by Yameogo et al. [28] presents a method for the prediction of cutting forces and chip morphology by using a 2D FE model. The authors establish a link between chip morphology and cutting forces level. Studies such these are important to have a better understanding of how certain parameters influence the generated cutting forces. In a study performed by Sreeramulu et al. [29], a FEM three-dimensional model was used to predict the cutting forces, as well as the other outputs of the turning of Al 7075-T6 alloy, using a coated TiC/Al_2_O_3_/TiN titanium carbide. The authors validated the information obtained by the simulation by using a dynamometer to assess the cutting forces, and they have also measured the machining temperature developed during the process. The values obtained for the simulation presented high accuracy to those obtained experimentally. Studies like these are important, as they develop ways to evaluate the machining process without the need to lose time and money in experimental procedures, especially when machining materials considered hard to machine, as the forces and temperatures developed during the machining process tend to be higher.

The FE method enables the understanding of the development of cutting forces in relatively new processes, such as high-speed diamond turning and milling, in which a study on these processes reveals that there are real advantages of these processes relative to standard diamond machining. A recent study has revealed that the machining time and cutting forces would be reduced when employing these processes over the standard diamond machining process, which would improve the cutting tools’ lifespan [30]. Nowadays, FEM is also being employed in the machining of composites. Some studies are using this method to assess the effect of tool wear on the machining forces of CFRPs (Carbon Fiber Reinforced Polymers). Moreover, these methods allow us to obtain useful information for developing new cutting-tool geometries for machining that can further improve the machining of these materials [31].

Regarding the cutting force prediction for machining composites, there are some recent papers focused on this matter. For example, Zhang et al. [32] presents a novel stochastic model of cutting forces in the milling of fiber reinforced ceramic matrix composites. These materials have seen great application in various industries, such as electronic and aerospace. The authors have modeled the cutting forces by combining the influences of randomly distributed carbon fibers and the stochastic aggravated tool wear simultaneously. They have identified that, unlike metallic materials, the forces generated during the machining of these composite materials is mainly developed in the shear deformed region, the friction deformed region and the ploughing region. The forces developed during this process are mainly caused due to the fiber cracking and fiber-matrix interface debonding, as well as elastic and plastic deformation of the material. The authors have validated this model by conducting milling experiments on these materials and measuring the cutting forces. The values obtained from the prediction agreed with the experimental tests; furthermore, the developed model can predict the cutting forces of the process, regardless of the machining conditions. Wang et al. [33] have developed a model based on ductile and brittle facture material removal modes for edge surface grinding of CFRP composite, using rotary ultrasonic machining (RUM). The cutting force is the main criteria to evaluate the performance of this process. The same authors have developed another model for the prediction of cutting force in the feeding direction [34]. However, in that paper [33], the authors claim that this model is developed for the prediction of cutting force in feeding direction and cutting force in depth-of-cut direction. The values obtained from the model have been validated through experimental tests. The authors have found that both the force components can be controlled by altering ultrasonic amplitude, tool rotation speed, feed rate, depth-of-cut, abrasive-grain concentration, or the abrasive-grain size.

Regarding the cutting force compensation, Wang et al. [35] propose a method for frequency response function (FRF) modeling based on transfer path analysis (TPA) and receptance coupling substructure analysis (RCSA). The FRF is required for cutting force compensation, as these forces are difficult to measure accurately, due to the dynamic characteristics of cutting forces’ measurement systems. The authors obtained a verified model which could be used for FRF prediction, conducting milling tests for algorithm verification. However, although the FRF of the workpiece can be obtained by FEM, for various geometries, the FRF of the dynamometer needed impact test results, to obtain the parameters. This method represents another effective way of predicting/controlling the cutting forces of a machining process. Although this method still needs improvements, it can be applied to both turning and milling machining processes, enabling its optimization.

As previously mentioned, cutting force knowledge can help understand the process’s details and enable its optimization and understanding, being of great use on machining cases of hard-to-machine materials. Regarding the prediction of cutting forces in the milling process, in the study developed by Tsai et al. [36], two methods of predicting the cutting force for the milling of Al 6060-T6 alloy are presented. These methods are as follows: Altintas and recursive least square (RLS). All the obtained values were experimentally validated. The authors investigated the influence of parameters such as, feed per tooth and tool diameter on the cutting force. It was concluded that the prediction obtained through the RLS method agreed with the experimental values for the forces; however, the Altintas method had certain peak values that did not match the experimental ones. The authors found that an increase in feed rate would result in an increase in cutting forces in the tangential direction. Cutting force determination on five-axis machining processes can be difficult, due to the changes in cutter orientation during the machining of complex parts. In the paper presented by Zhu et al. [37], the authors propose a cutting force prediction model by applying a new method, the cutting-edge element moving (CEEM) method, to calculate instantaneous undeformed chip thickness (IUCT) to effectively simulate five-axis machining cutting forces. Still regarding cutting force prediction in five-axis milling, in the study carried out by Olvera et al. [38], the authors propose a method of predicting forces in the milling of Al 7075-T6 alloy, using barrel-shaped cutters. These tools offer an advantage in the production of complex aircraft parts, offering chatter-free high-performance machining. The proposed model used the main geometrical parameters, orientation tilt, lead, and angles of the process, achieving a good agreement with experimentally obtained values. Studies such as these are quite important, as they pave the way for the testing and development of new tool geometries, highlighting the importance of these models on matters such as machining process optimization.

There are some recent developments in the prediction of cutting forces in micro-milling, a process where cutting force knowledge is of crucial importance. An analytical model was already developed for micro-milling operations, based on the process’s geometry, and considering the most relevant factors that influence the cutting forces [39]. In that study, it was found that, in micro-end-milling of steel and aluminum alloys, the parameters that had a greater influence on the cutting forces were tool run-out, tool deflection, size effect, and tool entry and exit angles. In the study presented by Zhou et al. [40], the authors present an analytical modeling of cutting forces in micro-end-milling of NAK80 steel, considering edge radius and material strengthening effects. The model presented is based on the classical oblique cutting model and the slipline model. The authors conducted experimental tests for the validation of the developed model, and the average error was determined for the X and Y directions, being 5.23% and 8.02%, respectively. Still regarding cutting force prediction in micro-milling, this study by Wojciechowski et al. [41] proposes a numerical–analytical model for the prediction of these forces during the micro-milling of AISI 1045 steel, considering chip thickness accumulation. The authors mainly studied the phenomena occurring in the burnishing and chip-formation-dominant regimes. The model considered the geometric errors of the machining system, the minimum uncut chip thickness, tool deflection, and chip-thickness-accumulation phenomenon. The authors analyzed the chip thickness accumulation during the process, which was an important source of machining force variations. These variations in force can affect the stability of the process. In another study performed by Zhang et al. [42], the authors propose a new instantaneous force model, in which the size effect is reflected in the cutting force coefficients, and the tool runout effect is included in the instantaneous uncut chip thickness. The engagements of the tool are identified together with the position of entry and exit. The theoretical forces obtained by using this model were compared with experimental values and were validated, with the maximum peak errors being no more than 0.6% of the experimentally obtained values. Studies on this matter also enable us to know the influence of the tool coating and tool geometry in the prediction of cutting forces, as described in the work carried out by Sahoo et al. [43], where the influence of TiAlN coating and limiting angles of the flutes on the prediction of cutting forces in micro-milling of P20 steel is presented. In that work, the authors used analytical models for the determination of cutting forces, and these models were applied by extracting force coefficients from FEM simulations for TiAlN coating. Data from an uncoated WC tool were also extracted and used for comparison. In this article, the authors prove the viability of this model, with cutting force values being predicted for the TiAlN-coated tools, with 16.26% and 15.84% for the X and Y directions, respectively. However, the error in cutting force prediction was significantly higher for the WC tool, being 33.15% and 29.56% for the X and Y cutting force directions, respectively. Studies such as these are crucial for understanding the dynamics of micro-milling, as well as its control.

Regarding cutting force determination in the turning process, the study performed by Orra et al. [44] proposes a mechanistic model for the cutting forces prediction in the turning of AISI 52,100 and AISI 4340 steels. The cutting forces are predicted with the information provided by chip morphology and nose radius influence on it. The method was successfully validated for these two materials. Furthermore, the model achieved a confidence level of 95%, accurately predicting cutting forces and providing a viable and economic way for the prediction of cutting forces, at least for these two steels. Kesevan et al. [45] studied the influence of cryogenic coolant during turning of Hastelloy C267 on the cutting force, thrust force, feed force, and cutting temperature. The authors carried out numerical simulations by using Deform 3D. Furthermore, the authors determined the optimal cutting conditions by using Lagrangian formulation. The authors verified that there was a 57% temperature reduction when compared to dry turning. Values obtained from the simulation were very close to the experimental values achieved, with the maximum error for the cutting temperature and cutting forces being lower than 5%. A physically based model for cutting force and microstructural modifications in Inconel 718 was developed by Rinaldi et al. [46]. The authors used FE method to determine the microstructural modifications occurred during turning process, by simulating different strain, strain rate, and temperature conditions. The authors verified that predicted cutting forces and the measured forces were close, with the error being lower than 5%, proving that this model is viable. As mentioned in the Introduction, one of the methods commonly used to predict cutting forces is the Kienzle Force Model [5,18]. In the study performed by Salehi et al. [47], using extended Kienzle force model, the authors propose a prediction method of cutting and ploughing forces in the turning process. The prediction of these forces was performed by using nonlinear least square fitting and Bayesian inference methods. It was found that the Bayesian inference can predict cutting and ploughing forces with minimal initial data, which means that this method is a preferred approach when the initial data are very reduced and there is inherent uncertainty. In the recent study presented by Toubhans et al. [48], a cutting force model for the turning of Inconel 718 was developed, which enables the prediction of cutting forces and their evolution along the tool pass, for a wide range of surface finish levels of quality. The authors found that including a component relative to the chip flow direction in the local force model allows for better force predictions. The tool wear, in this case, has a critical impact on cutting forces, with these increasing with the increase in tool wear.

These methods prove to be a low-cost approach when compared with current commercially obtained dynamometers. However, these methods also present some drawbacks, such as time constraints and the need to know the proper inputs, especially in case of simulations. Numerical–analytical methods are frequently paired with other cutting force measurement devices, to adjust force measurements, making these readings more accurate. They complement force measurement systems, obtaining a more detailed information on cutting parameters and tool health, enabling the optimization of these machining processes, either by improving surface-finish quality, tool life, or even cycle time.

**Figure 1:**
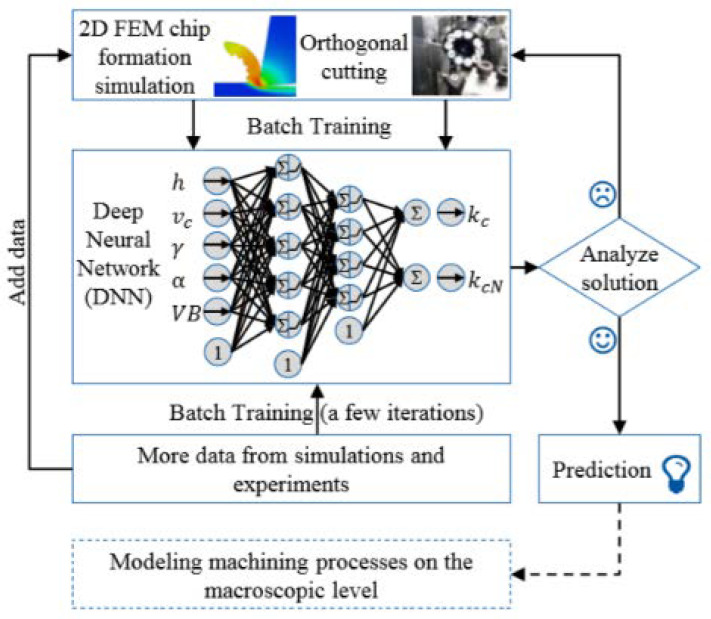
Hybrid approach on machine-learning architecture [51].

Machine Learning Methods are also being employed in the determination/prediction of cutting forces. Generally, a machine learning model is utilized to acquire information from various sources, such as machining operations (milling, turning, etc.) and to obtain data from numerical methods, such as the finite element method. This enables the prediction of specific cutting forces on similar operating conditions. Machine learning can also lead to cost and time savings, increased quality, and material waste reduction [49]. In the work performed by Wenkler et al. [50], it is described the possibility to record and predict cutting forces from a milling process. Data are obtained from a monitored milling process; these data values are collected in a database. An Artificial Neural Network (ANN) is trained to predict the specific cutting force. If predicted forces have a large deviation, a control loop ensures that the ANN is relearned with the extended data basis. This enables the evaluation of a capability of a machine to execute, before performing its task. The authors also state that this model should be used as a base for further analysis of different cutting processes, such as, drilling and turning [49]. Another paper presents a hybrid approach of data collection, when using machine learning to predict cutting forces, considering tool wear in a turning operation [51]. Here, data are collected from both a dynamometer and a 2D FEM model, which enables this machine learning model to predict not only cutting forces but also tool wear, thanks to the 2D FEM model, as seen in the model depicted in Figure 1.

These methods are still quite new and very dependable on a reliable database. Moreover, they are still quite slow when it comes to cutting force prediction mainly due to the data collection, specially data from FEM models, as these are very time-consuming. However, machine learning methods show a very promising future, predicting the best machining parameters and tool wear, and thus boosting the machining process’s productivity.

#### Research Trends in Cutting Force Prediction

Here, the recent trends of predictive methods in cutting forces are going to be presented and analyzed, based on the collected articles presented in the last section. The main advances made in prediction methods and their advantages are going to be mentioned, highlighting the possibilities that some new methods bring regarding the cutting force prediction.

The growing knowledge on machining processes and immense amount of data experimentally achieved has caused an improvement on the predictive methods, which are usually dependent on these data. New models and methods for cutting force prediction have been developed and validated, being now considered quite reliable. The developed and adopted models not only are effective for cutting force prediction, being also reliable for the prediction of parameters such as surface roughness, tool temperature, and even tool wear. This is reflected in the common adoption of FE methods for machining process simulation, as these enable cutting force, tool/workpiece temperature prediction and tool wear throughout the machining process.

Analyzing the work performed by the various authors, we see the main focus is on the improvement of already existing prediction methods and models, as well as the creation of new prediction methods and models for various machining processes. The work seeks to overcome common problems in the cutting force assessment field, such as high cost of the cutting force system or complex part cutting force measurement/prediction. In Table 1, the main research topics, their advantages, and possibilities, as well as the main drawbacks found in the research, are presented.

### 2.2. Measurement of Cutting Forces

The cutting forces measurement can be made by using various methods, such as using a dynamometer, a cutting force sensor, or by obtaining cutting force values based on various machining factors, such as tool deflection or vibration [52]. In this section, various ways of measuring cutting forces are presented, based on a collection of recent researches on this matter.

As previously mentioned, the monitoring of cutting forces is of great importance, not only to improve/optimize the machining process, but also to gain insight into the cutting behavior of some tools, or even achieve knowledge on the machining process itself. Furthermore, cutting force determination/monitoring can bring the improvement of some cutting-tool technology, such as the use of new types of coatings for cutting tools [13,14], or even studying the influence of novel geometries, and how these will affect the cutting forces generation.

**Figure 2:**
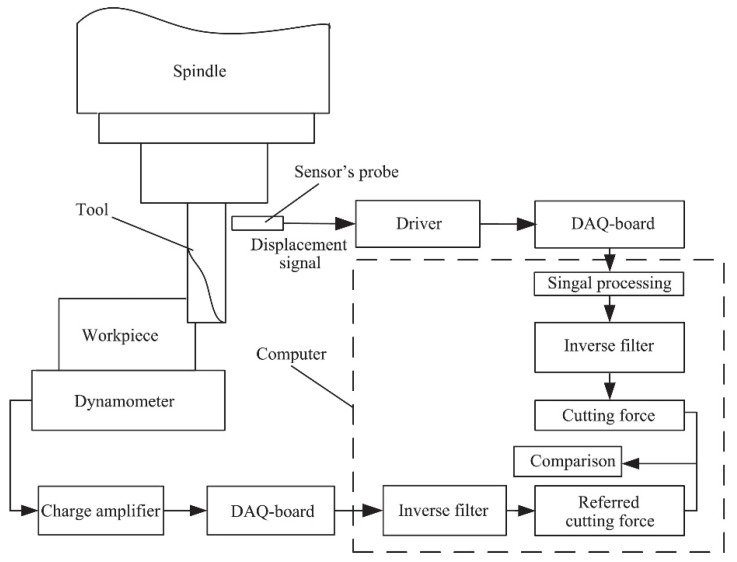
Experimental procedure for the measure and comparison of cutting forces during machining [53].

Cutting force also can be determined by using some machining parameters to calculate them. Depending on the cutting force, some examples of these parameters are spindle motor current, voltage, acoustic emission and vibration [17]. In the case of a recent study on indirect cutting force measurement [53], cutting force values are achieved based on tool flank deflection. A displacement sensor is attached to a tool shank and used in a cutting operation. The datum collected is the deflection signal, which is then filtered and used to calculate tool stiffness, being usually used in turning processes to obtain the cutting force values. This is very important, as obtaining reliable readings of cutting forces is very useful for the selection of proper machining parameters and tool geometries. The cutting force values were compared with the readings of a dynamometer (Kistler 9255B), using the experimental procedure presented in Figure 2. It was concluded that the method presented by the authors is reliable in obtaining the cutting force values. This method can be applied to a workpiece of any size.

**Figure 3:**
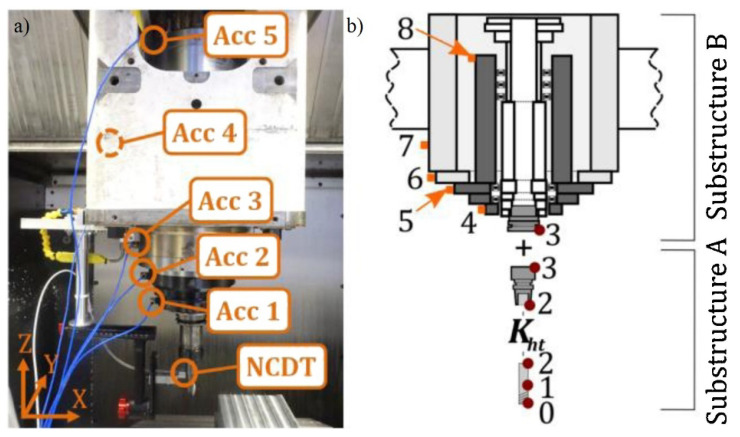
(**a**) Real image with the position of the five accelerometers and one non-contact displacement transducer (NCDT) indicated in the machine’s spindle. (**b**) Scheme of the spindle (divided into two substructures) with 8 positions indicated for the calculation of the Frequency Response Function [55].

Still regarding the calculation of cutting forces by measuring a machining parameter, Miura et al. [54] developed a method of estimating cutting forces by using an external power measurement system. The induced voltage is calculated by measuring three-phase voltage and current signals of the feed-drive motor. The calculated voltage is then multiplied by the corrected current, which is done in order to obtain the cutting current. The authors have conducted two-dimensional milling tests for the validation of the process. This method is a promising one when it comes to low-cost cutting force measurement; however, it still needs further validation. Another method for the estimation of cutting forces is proposed by Postel et al. [55], by mounting accelerometers on the spindle housing, during milling tests performed on Al 7075 alloy, with a two-fluted end-mill that is 20 mm in diameter. The accelerometers were mounted as shown in Figure 3.

The authors have adopted the configuration shown in Figure 3, as it enables for a more complete data collection, as they state that only one accelerometer per direction would be enough to determine the cutting forces and vibrations developed during the process. This method was validated by performing milling tests and using a dynamometer (Kistler 9265B) to measure the cutting forces. For a more accurate cutting force estimation, a Kalman filter was employed to compensate the cutting force values, enabling the modeling of the proposed system.

Another way of measuring cutting forces is by employing sensors, such as dynamometers. These sensing methods are those that employ effects that are directly related to the cutting forces, such as mechano-magnetic, mechano-electric, and mechano-optic conversions [17]. These sensors range from optoelectronic and piezoelectric dynamometers to strain gauge and capacitive force sensing systems. They can be employed on their own, registering data regarding cutting forces that enable the understanding of the machining process of different materials and even the determination of optimal parameters, for cutting force reduction and tool-wear characterization [56,57]. These can be paired with various numerical methods or simulation methods, to provide a more complete view on what is happening during machining [58], or to correct the measurements of these devices. With the knowledge of the cutting forces provided by these devices, the optimization of certain machining processes and the machinability of certain materials becomes easier.

There are some recent developments on dynamometer types, such as a novel triaxial optoelectronic-based dynamometer presented in the Subasi’s work [59], in which the authors describe the development of a compact and triaxial dynamometer which uses photo-interrupters. This dynamometer can detect forces in three orthogonal dimensions and presented as an alternative to current dynamometers, being a compact and relatively low-cost solution. In another paper [60], a displacement-based dynamometer for milling force measurement is proposed. A flexure-based strategy was implemented in the milling setup, where structural deconvolution was carried out by filtering the measured displacement signals. Cutting force measurements were successfully obtained when using this approach, for both Single Degree of Freedom and Multiple Degrees of Freedom flexure-based dynamometers. Otherwise, Sandwell et al. [61] present the development of a multi-degrees of freedom optical table dynamometer. In that paper, the development and implementation of an optical force dynamometer is described. A four-quadrant position sensitive photodiode is used to detect the position of a target surface to estimate the applied force in three independent directions. The use of this single detector minimizes the overall cost of the system. Multi degrees-of-freedom measurements are permitted without significant loss due to the implementation of a novel decoupling algorithm and oscillator detector scheme.

In the work carried out by Transchel et al. [62], a dynamometer for the measuring of force signals in the micro-machining operations is presented. The sensor presented is a piezoelectric dynamometer (MicroDyn). Due to the high rotational speeds that are characteristic of the micro-machining process, there is high excitation frequency. This frequency interferes with cutting force measurement, making it impossible with regular commercially available dynamometers. The authors have tested the dynamometer, which enables the measurement of forces in high dynamic cutting process with frequencies up to 5 kHz.

**Figure 4:**
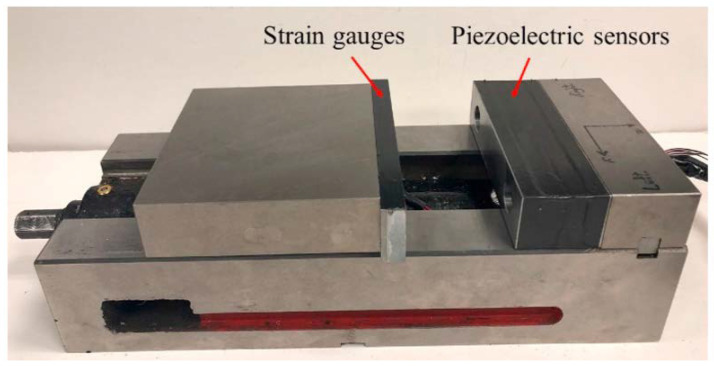
A prototype integrated with jaws embedded in strain gauges and piezoelectric sensors [65].

Regarding the development of dynamometers, in the work developed by Totis et al. [63], the development of a table dynamometer for advanced milling and drilling applications is presented. The dynamometer was designed for precise milling and drilling, with small cutters and at high spindle speeds. The device can measure the three resultant components of cutting force. Additionally, the dynamometer can measure cutting torque in the drilling operations. This device has an extended range of measure when compared to the most accurate dynamometer table commercially obtained. Furthermore, this device has an excellent dynamic behavior, i.e., it can measure cutting forces accurately at relatively high frequencies (generated by high spindle speeds). In another study, the development of an integrated rotating dynamometer on a tool holder is presented by Rizal et al. [64]. The developed dynamometer was a strain-gauge-based rotating sensor, directed primarily to the milling and drilling processes. The novelty of this dynamometer comes from the fact that the tools are interchangeable, which are compatible with different standard modules, making this a very flexible system, with easy reconfiguration. The device measures force up to 3000 N, and it supports rotations up to 5000 rpm. The authors report that the cross-sensitivity errors are below 4.05%, which is an acceptable value. This dynamometer can be applied to cutting process optimization, machine-tool monitoring, and design. This system, coupled with the high flexibility of this device, makes it very appealing. Another interesting and recent development of a cutting force measuring system is presented by Rezvani et al. [65], where a force sensing system, consisting of a vise with built-in piezoelectric and strain gauge sensors is used. The authors present a prototype, shown in Figure 4, with six piezoelectric sensors for the measurement of dynamic forces in the X, Y, and Z axes. The position of the strain gauges in the system was selected by calculating strain values, using FE simulations.

However, the authors encountered problems with limitations in bandwidth, which negatively impact the performance of the sensors [65]. To correct it, the authors have implemented a Kalman filter as a force-compensation method. There are also some limitations regarding the cutting temperature of the tests. Although there are some limitations, that work presents a novel and interesting concept for cutting force measurement. Still regarding the development of novel cutting force measurement methods, Zhao et al. [66] present a cutting force sensor based on semi-conductive strain gauge as a way to overcome some problems in cutting force measurement such as sensitivity, natural frequency, compatibility and the high cost in recent cutting force sensor developments. A new sensing element was developed, consisting of two mutual-perpendicular octagonal rings, which was done to improve natural frequency. The authors conducted preliminary tests regarding, static calibration and modal impact. Furthermore, a dynamic cutting experiment was also conducted on a lathe, and cutting forces were measured. The results from these preliminary tests proved the feasibility of the proposed system. The semi-conductive strain gauged fabricated by MEMS (micro-electro-mechanical system) showed an improvement in sensitivity by 16 times comparing to a traditional strain-gauge-type sensor. This system showed promising results in the measurement of dynamic cutting forces. Although this system is very promising, there is still room for improvement, as stated by the authors. Future research will focus on the sensor structure optimization and overcome the problems regarding the measuring of cutting forces in high-speed machining.

The importance of measuring these forces is well-known and provides the ability to understand the behavior of tools and materials during the machining process, by analyzing the wear behavior and the impact of this wear on cutting forces, or even enabling the evaluation of the machinability of certain materials [67]. The latter can be understood as very interesting when it comes to the machinability of hard to machine materials, such as some titanium alloys or even composites. In the work developed by Palasinamy et al. [68], the wear influence of a TiAlN coated tungsten carbide insert during turning of AISI 420 steel is evaluated. It was found that the radius of the tool’s nose, feed rate, and cutting speed have direct influence on the cutting forces during machining, with lower feed rate and tool-nose radius, and higher cutting speeds being preferred. The cutting forces were registered by using a Kistler dynamometer (Type 9257B). Regarding machinability studies, the study recently conducted by Pandey et al. [69] focused on the machinability of the nickel-based superalloy Inconel 825, which was evaluated during dry turning, using an uncoated WC-Co tool. The authors found that the cutting forces raised up with cutting speed, increasing 20% in tangential force when increasing the cutting speed from 70 to 200 m/min. In this study, the wear of the coated tool was also analyzed, being reported as main wear mechanisms the abrasion and adhesion. Still regarding machinability studies, Kumar et al. [70] conducted a study on the machinability of Ti-6Al-4V titanium alloy during dry turning, using cryogenic treated and untreated uncoated WC inserts. The authors reported that, during the turning of these titanium alloys, the thrust force is the most dominant, over feed force and cutting force. The machining parameters showed the principal influence on the cutting force, although the wear was less evident on the cryo-treated WC inserts.

These dynamometers can also be applied in studies related to machining lubrification. In this case, a study [71] was conducted for a turning operation of a low carbon steel (St52-3), using a multi-component dynamometer (Kistler 5070) to gather the cutting forces generated during the process. The tests were conducted under Minimum Quantity Lubrification (MQL), and the authors concluded that, under this condition, the machine consumed less power. In addition to the low quantity of lubricant used, this means that this lubrification method has some advantages when used for the machining of low carbon steel. In another study of the influence of cutting fluid on machining forces, Sharma et al. [72] measured the machining forces developed during the turning of AISI304 steel, using alumina-multi-walled carbon nanotube (MWCNT) hybrid nanoparticle enriched cutting fluid. The machining forces were measured by using a three-component piezoelectric Kistler dynamometer (Type 9047CNK). The authors reported that using this fluid effectively led to cutting force reductions by 20.2%, 21.3%, and 13.6% in the X, Y, and Z directions, respectively. Another study on the influence of cutting fluid was conducted by Oliveira et al. [73]. They investigated the influence of cryogenic cutting fluid, MQL, and flood methods during end milling of Inconel 718. The cutting forces were measured by using a dynamometer, Kistler 9257 BA, with a signal amplifier and an A/D data acquisition board. The authors found that the resulting cutting force levels were very similar for flood and MQL method. However, these forces increased by 15% when using liquid nitrogen (cryogenic cutting fluid).

In order to develop tools that perform better and longer, the manufacturers study tools’ cutting behavior. Dynamometers are employed in the study of the behavior of these tools in various machining processes. The knowledge of these forces is also beneficial to the understanding and development of new coatings. Some of these coatings are employed to reduce cutting forces, improving the surface roughness of the workpiece, and increasing the tool’s lifespan [74,75].

**Figure 5:**
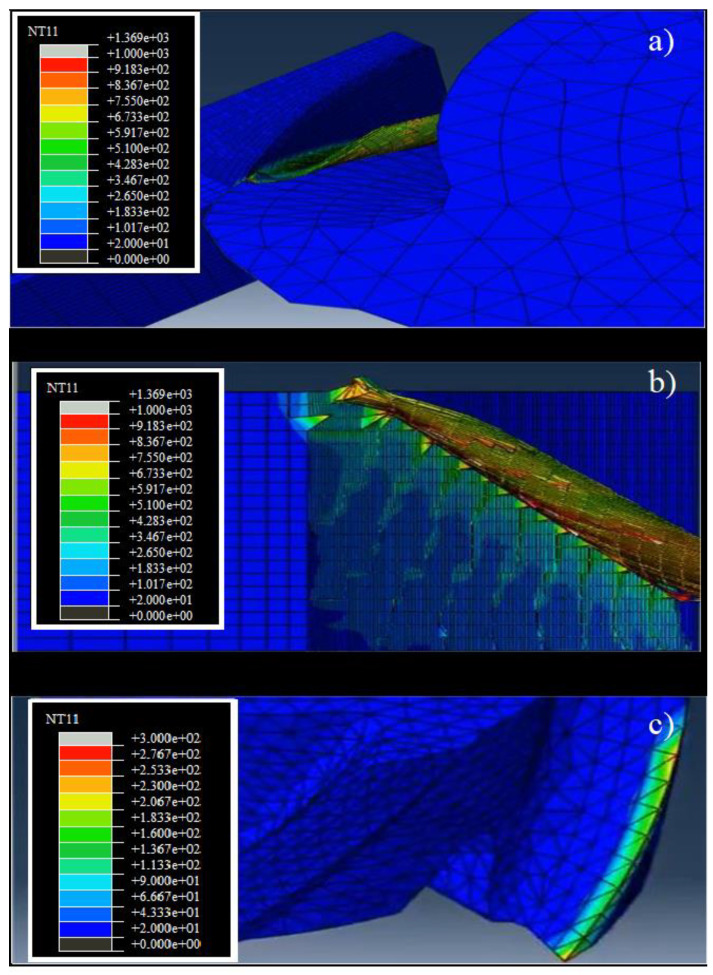
Temperature distribution on the tool–workpiece interface (**a**), workpiece (**b**), and tool (**c**) [76].

There are also cases where a numerical method is used to correct the signals obtained from cutting force sensors, as in the study of cutting force measurement correction obtained with a table dynamometer. Frequencies from the machining process can interfere with the readings from the dynamometer. Thus, Wan [11] proposes the use of a Kalman filter to correct the force measurement, achieving a successful implementation. The data may also be insufficient, and then a hybrid measurement method can be employed. These methods are coupled with force sensing systems, to give detailed information impossible to be provided by the dynamometer, e.g., tool temperature. The work performed by Daniyan et al. [76] presents a solution for optimizing the milling of AISI P20 steel. In that study, the authors employed a dynamometer to collect cutting forces during the milling of that material. Process simulation was carried out by using Complete Abaqus Environment, to know the temperature distribution over the tool (Figure 5c), the workpiece (Figure 5b), and the tool–workpiece interface (Figure 5a) during machining.

Additionally, Response Surface Methodology was employed to study the interactive effects of the cutting speed and feed rate. With all this information, the authors created a way to optimize this milling process, achieving a better material removal rate and surface finish, if the feed rate and cutting speed parameters are changed.

Regarding methods that employ both analytical processes and force sensor methods, Kene et al. [77] present a study about the development of an analytical model for the monitoring of tool health in hard machining. The model uses data from three sensors: the cutting force (provided by a dynamometer), the tool vibration (provided by an accelerometer), and the surface roughness (surface roughness tester). Furthermore, the optimal machining parameters (cutting speed, feed, and depth of cut) were determined and used. All of this was used to create a fusion function that can be used to predict the tool’s wear, with an error percentage below 9%.

The papers mentioned previously [59,60,61] state the importance for a cost-efficient alternative to the force sensing systems. Dynamometers provide mostly accurate cutting force measurements, that can be obtained very quickly when compared to the numerical–analytical methods, which, in turn, enables the faster analysis/optimization of a machining process. The accuracy problem of these measurements can be corrected, as stated previously, using numerical methods. However, the acquisition cost of these devices is still quite high, adding a barrier of entry, especially in this field of research. With the monitoring of cutting forces being so important for process optimization or tool lifespan monitoring, there is a need for a lower-cost alternative to this equipment, so that more research can be performed. Another drawback besides the high cost is that these dynamometers interfere with the system where they are implemented, i.e., the device stiffness and assembly itself affect the cutting force measurements, so there is the need to implement some form of correction method, by figuring out a better setup (that would not interfere with the cutting force readings), or by developing new types of force sensors, with the aim to minimize these problems.

#### Research Trends in Cutting Force Measurement

Cutting force measurement, as seen from the previous section, is of great importance for the development of new more effective methods for machining certain materials.

The work presented is mainly focused on a few points:

1. Employment of dynamometers and cutting force sensors to measure and analyze the cutting forces of processes that are not yet fully understood;
2. Development of dynamometers and sensor systems that enable the measurement of cutting forces in more complex machining processes;
3. Development of a low-cost alternative to current commercial measurement systems;
4. Improvement of cutting force reading, using analytical/numerical methods paired with measurement systems.

Cutting force knowledge is key when desiring to optimize a machining process, because there is always a need for improvement of cutting force measurement systems and for the creation of new, more competent machining methods and tools. Cutting force measurement methods are commented on in the last section of this paper (Section 3).

The main advantages and drawbacks of each of these research topics are presented in Table 2.

### 2.3. Cutting Force Determination in Robotic Machining

Robots are already implemented in industrial machining operations that involve relatively low cutting force requirements. Uhlman et al. [78] studied the usability of industrial robots for trochoidal milling, a process with relatively low cutting forces; however, this matter still requires some more research, because this is still considered unviable. Robots bring many advantages in the machining area; they can be implemented with analytical or numerical models to improve tool path accuracy. Sensors can be easily implemented on a robot, making these robots adapt their actions depending on the sensor data. The opportunity to implement machine learning models is possible because robots can learn and adapt their behavior, depending on the data acquired throughout the machining processes. Robots may even present a lower cost, in the long-term, when compared to machining centers. This, coupled with the opportunity to improve machining accuracy for robots, gives them a significant advantage in machining tasks. Additionally, robots are flexible and able to perform different machining tasks [79]. There are some papers about using robots for the determination of cutting forces. As stated previously, sensor implementation on robots is quite easy, and there is a need to study the effect of the robot dynamics in the machining processes yet. The work performed by Cen et al. [80] studies the effect of robot structural vibration and cutting forces in a robotic milling process. Studies like this are a necessity for a better understanding of the forces developed in robot machining, as the easy sensor implementation in robots and their autonomy to correct and adjust the process reveals promising applications in the area of the machining processes. Another work carried out by Rivière-Lorphèvre et al. [81] proposes a 2D1/2 model approach to compute the cutting forces in milling if the cutting-tool axis is tilted. This enables a more accurate prediction of cutting forces for multi-axis machines or robotic machining.

In the study carried out by Slamani et al. [82], the effects of machining parameters on cutting force components were studied during high-speed robotic machining of CFRPs. The authors created a model for the prediction of cutting force components regarding different cutting speeds, feed, and robot configuration. The model was then validated based on the experimental data. The authors report that the created models are valid in the prediction of cutting forces for this application. Studies like this enable us to calibrate the machining process, to optimize the cutting forces generated. The reduction of this leads to an overall better-quality work. In that case, it avoids the delamination of the CFRP. Furthermore, it reduces the robot deflection and the tool wear suffered during the process. The robot configuration is also very important, including the number of joints, or the motion of the robot. With the cutting force knowledge, this can also be optimized in order to achieve the desired results, such as optimal robot configuration, optimal task placement zones (based on robotic arm reach), and robotic machining motion, which can lead to lower cutting forces [83,84].

Due to the robot arm dynamics during its functioning, cutting force measurements are negatively influenced. In the study performed by Nguyen et al. [85], an inverse filtering method is proposed as a way to correct the influence of the robot arm dynamics. The authors use a GPR (Gaussian process regression) approach to predict the FRF of the flange force sensor. They report that this method improved the accuracy of the flange force sensor’s measurements. Furthermore, this method can be applied to other robotic applications requiring dynamic force sensing.

Robotic machining reveals a great potential relative to work productivity, tool-wear optimization, and overall machining time reduction. The ability to easily implement sensors on robots enables for a better interference-free cutting force measurement in machining processes. Robots’ ability to correct in real-time machining parameters and learn (when coupled with machine learning models) as they work provides an attractive alternative to the traditional CNC machining processes. Currently, numerical methods are being developed to predict cutting forces in multi-axis machines and robotic machining. However, these still need validation, and cutting force measurement is still mostly achieved by using dynamometers implemented in conventional machining setups.

### 2.4. Process Optimization Using Cutting Force Data

The optimization of the machining process is still a very important problem, and there is a lot of research on the improvement of these processes (machining), determining the best machining parameters to the improvement of tool lifespan. Cutting force data, which is obtained during these processes, is of high importance regarding the process’s optimization, as the cutting forces developed during machining provide an insight into what is happening during the process, thus enabling its tuning for the required goal. Some common goals in the machining industry are improving surface roughness quality, material removal rate, and toolpath, with the latter being very relevant to the milling of complex parts [86]. Cutting force monitoring and optimization on a machining process can also be used for the optimization of tool lifespan [87], by monitoring the tool wear in machining processes as they progress. This proves to be very useful for the knowledge of the process and tool behavior [88].

**Figure 6:**
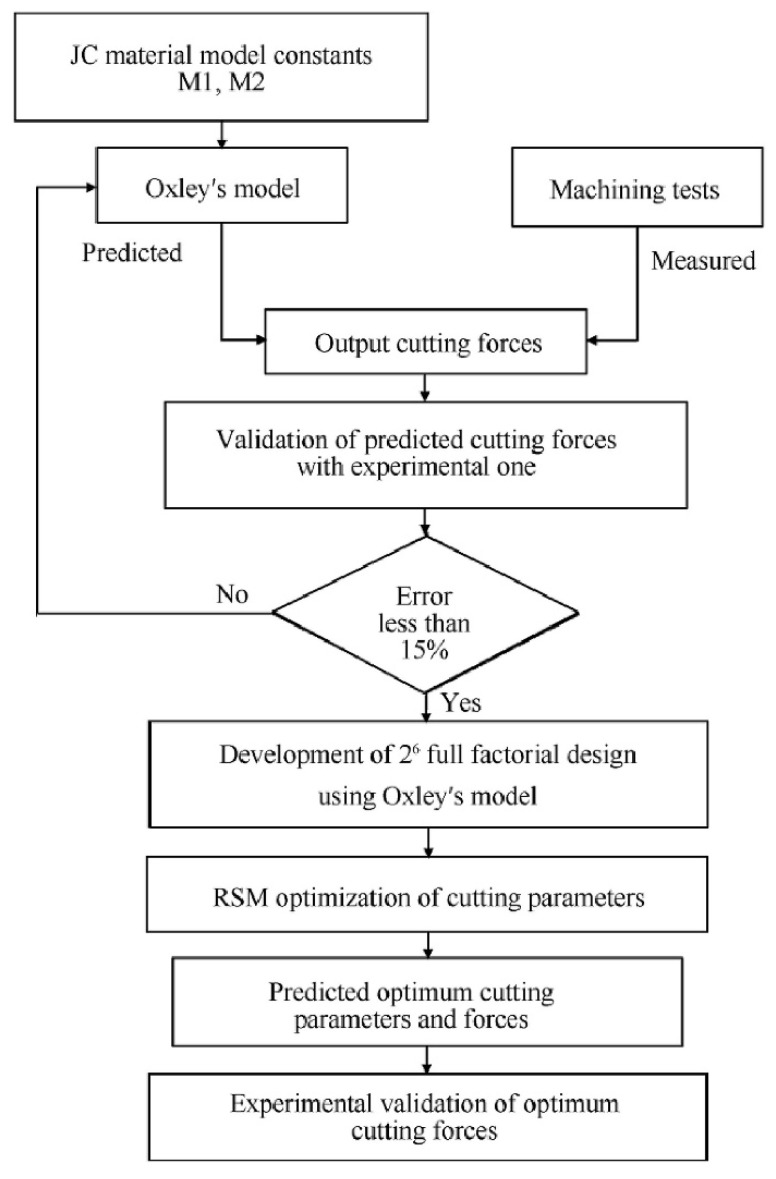
Scheme of the methodology adopted for the prediction and optimization of cutting forces [89].

Regarding cutting parameter optimization, using cutting force prediction, the paper presented by Sagar et al. [89] proposes a method for the prediction and optimization of cutting forces, using Oxley’s predictive theory and the Response Surface Methodology (RSM), in the machining of Tungsten heavy alloys (WHAs). The authors employ these methods to predict the cutting forces at different feed and speed combinations. The schematics of the methodology used by Sagar et al. [89] can be observed in Figure 6.

The presented analytical cutting force model was developed with success, which was validated by comparing the obtained cutting force component’s value with cutting force data obtained from experiments. It was found that, in the presented case, the most influential parameter on the cutting forces was feed rate. The authors also determined the optimal values for the machining parameters, in order to minimize the cutting forces developed during machining.

Numerical or mathematical methods have also been employed in the fine-tuning of cutting force measurements obtained from force sensing systems. The data collected by these devices can be incorrect due to certain parameters such as complex geometry of the cutting area, outside interferences, and even machining frequency. There are studies such as the one presented by Venkatesan [90], which employed the Taguchi Method to determine the optimal machining conditions in a laser assisted machining process of Inconel 718. The data regarding cutting forces are collected by a dynamometer. The authors optimized the process, focusing on the decreasing of cutting forces during the material machining. This study highlights the potential of cutting forces knowledge in these machining processes. There is some recent research made in the study of cutting forces in the ultrasonic assisted milling process. In the study carried out by Verma et al. [91], a statistical model is presented which evaluates the effect of process parameters, on the cutting forces generated during the process and the effect on the cutting force deviation. The evaluated parameters were the power of ultrasonic vibration, rotational speed, axial depth of cut, and feed rate. Milling tests were conducted on Al6063 alloy, and then an ANOVA was performed to obtain the regression equations. The results obtained from the milling tests were analyzed to study the effect of the parameters on the cutting forces. The authors validated this model and found that the parameter that influenced the most the cutting forces was feed rate. However, the parameter that influenced the cutting force deviation was rotational speed. Additionally, the authors determined the best parameters, in order to obtain the lowest cutting force values during the machining of the aluminum alloy.

Regarding machining process parameter optimization, Daramola et al. [92] performed a work aiming at the optimization of the machining of Ti-6Al-4V, using a piezoelectric dynamometer to study the cutting forces developed on the process. The goal of the authors was to minimize the resulting cutting force during the machining of this material. Thus, they were able to successfully set parameters on how to reduce the cutting force during machining, based on those results. In another recent study regarding machining process optimization, more specifically, cutting force optimization, Daiyan et al. [93] measured and optimized the cutting forces of a milling process of M200 tool steel, using RSM and a dynamometer. The used dynamometer was a Kistler 9257A 8-Channel Summation of Type 5001A Multichannel Amplifier. The authors managed to optimize the process, stating that the cutting force increased under dry milling conditions. It was also reported that the force sensors showed high levels of precision, detecting small values and variations of the cutting force. The study presented by Aslan [94] describes the optimization of a turning process of AISI 5140 steel. The optimization was made in terms of flank wear, cutting forces, and vibration. The author identified depth of cut and feed rate as being the most influential parameters for the cutting forces. Optimization of the cutting forces, flank wear, and vibration were carried out, using RSM, which enables the obtention of optimal turning parameters. This was validated by experimental turning tests with satisfactory results, being for the tangential force and feed cutting force 95.3% and 88.1%, respectively. Although the relationship studied in that paper has already been documented, this method provides a systematic approach that enables the optimization of parameters such as these. These kinds of studies are very valuable regarding process optimization, being directly related to productivity increase and improved component quality.

There have been some recent studies on optimization of machining cases of Ti-6Al-4V, as this material is still widely used in the aerospace industry, especially for structural components. In the study performed by Samsudeesadham et al. [95], the authors study the influence of machining parameters in cutting force. The authors conducted milling tests at a constant cutting speed of 40 m/min and found that depth of cut had a significant effect on the cutting force in the X and Y directions, whose are directly related to material removal rate. However, this parameter presented a very low influence on the cutting force in the Z axis. The authors registered a stable rise of cutting forces with the increment of feed rate until 0.06 mm/tooth, where a sudden rise in the cutting forces occurs. They also conclude that feed rate has a more substantial effect on cutting forces when compared to depth of cut. Tlhabadira et al. [96] present the development of a model for the optimization of energy consumption during the milling of Ti-6Al-4V. This is an important study, as the improvement of the cost effectiveness and sustainability of machining processes is a recent focus of the machining industry. They used RSM and determined the optimal combination of parameters (in terms of energy consumption). Cutting force values were obtained by using a Kistler dynamometer (9257A 8-Channel Summation of Type 5001A Multichannel Amplifier), and these forces are important, as they are directly related to the machine’s energy consumption. They were successful in determining optimal parameters for the milling of this titanium alloy, effectively minimizing the energy consumption of the process.

Tool monitoring is also an important aspect when it comes to process optimization, having to frequently replace machining tools and experiencing quality degradation problems as the wear of the tool progress are still problems in the machining industry [97]. This might also provide a valuable insight on the creation of new designs for machining tools [98]. In the work performed by Temsamani et al. [99], a tool-wear monitoring system for composite drilling is presented. They propose a line vision system with a high frame rate and a wide spatial coverage reconstructing alloying for the instantaneous 3D reconstruction of the wear profile of the cutting tool. The presented technology also enables the storing of cutting data, such as cutting force. This, coupled with the wear-out profiles, can provide valuable information on the assessment of cutting force data and tool wear for other cutting processes. Moreover, regarding tool monitoring, Urbikain and Lacalle [100] propose monitoring tool for the complete machining process, using Labview programming. The developed software was built from the combination of reconfigurable Input/Output (I/O) architecture and Field Programmable Gate Arrays (FPGA). This method reveals itself to be quite versatile, allowing for the recording of machining forces, accelerations, and acoustic pressures simultaneously, with the ability to register more variables such as machining temperature and displacement. All the obtained data can be stored and used later for further improvements of the machining process, which is very appealing for industrial applications. However, the proposed software requires a large quantity of accessories, as well as software licenses, which highly increases the cost of application of this method.

Cutting force measurement and analysis can also be used to compare coating performances and, thus, choosing the best suited coating for each application. For example, Gupta et al. [101] performed a study in which the cutting characteristics of three PVD (Physical Vapor Deposition) coatings are analyzed and compared in the turning of C45 steel. TiN, TiAlN, and AlCrN were the analyzed coatings. Due to cutting force information and hardness measurement, a relation between cutting force and depth of cut was established, and the coating with the best wear behavior was determined. The TiAlN coating, with its lubricating ability, lowered the cutting forces generated when turning the C45 steel. Studies like these highlights the importance of cutting forces’ knowledge. However, this can be used not only for process parameter optimization, but also for tool-coating selection. Moreover, regarding the optimization of cutting forces in machining operations using coated tools, Patel et al. [102] developed an experimental investigation on the end milling of AISI D2 tool steel, using AlCrN coated tools. In that work, 30 experiments were conducted. The cutting forces developed during the process were measured by a piezoelectric dynamometer, Kistler 9272. The authors have also used RSM to design the experiments. They have reached the optimal parameters for that case, in terms of cutting speed, feed, depth of cut, and width of cut, stating that the cutting speed and width of cut are the two main parameters influencing the cutting forces.

As seen previously, machine learning can be employed in the prediction of cutting forces; however, there have been some developments on the employment of these machine learning (ML) models in the optimization of the machining process, using cutting force data. In the study accomplished by Gouarir et al. [103], a system for tool wear prediction based on ML techniques and force analysis is presented. This system uses a force sensor to monitor the wear progression of the tool flank. Furthermore, it uses ML with a Convolutional Neural Network (CNN) to predict tool wear. The tests conducted were the milling of a stainless-steel workpiece, using a ball end mill, in dry machining conditions. The ML model uses a database which contains information of previous experiments. They report that CNN can identify the correlation between the cutting forces and the tool flank wear, achieved without any filtering methods. If the data are available, the prediction of tools’ wear can be made for any tool and any material. This also means that the effectiveness of this method is dependent on reliable data. Furthermore, the cost of this method is very high, especially due to the sensors utilized in the cutting process. However, it is a very simple method to implement, providing highly accurate predictions.

#### Analysis of Machining Process Optimization Methods

As previously stated, cutting forces developed during machining are tied with various parameters; thus, knowing and controlling these forces is the key to machining process optimization.

In the previous section, the presented articles mentioned focus on the optimization of machining processes, relying on the control of cutting forces, in order to obtain the desired results, as follows:

1. Lower cutting forces;
2. Lower surface roughness of the machined parts;
3. Lower energy consumption of the machines in operation;
4. Improve material removal rate;
5. Improve tool lifespan.

These optimizations can be achieved by various methods, either by employing an analytical method or an experimental method. There is a trend of using RSM to determine the optimal parameters for the desired result, usually coupled with a measurement system, such as a dynamometer, to determine the cutting forces developed during the process. To ascertain these forces, prediction and simulation methods can be employed. These two main cutting force assessment methods have their own advantages and drawbacks, which are addressed in Section 3.

In terms of process optimization, the analyzed proposed methods for the obtention of the aforementioned desired results present some strengths and weaknesses. In Table 3, the recently used optimization methods for machining processes are presented. Although machine learning models are a way of cutting force prediction, the developed models show great potential, as they are very versatile in terms of machining process optimization. Due to their novelty, these are later mentioned in the summary section (Section 3).

## 3. Summary

Cutting force assessment is a crucial research topic because it is very important for the understanding of the machining process, providing many advantages in terms of process optimization. These forces can mainly be obtained by two methods, either by employing a predictive method or by calculating/measuring these cutting forces. Articles regarding these assessment methods are presented in this paper. In Table 4 and Table 5, the main advantages and drawbacks of these predictive and measuring methods are summarized. Table 4 presents the common advantages and drawbacks of the presented methods for cutting forces prediction.

Although there are recent studies that show prediction methods providing high confidence rates, there are others that still require proper validation and must have a cutting force sensor to confirm the obtained values. Albeit, these methods look very promising. Indeed, with more reliable data being collected to strengthen some of these models, the cutting force prediction accuracy keeps growing.

Table 5 presents the common advantages and drawbacks of the cutting force measurement methods previously presented.

Cutting force data are still mostly achieved by using dynamometers and force sensing systems (namely piezoelectric dynamometers), despite their high acquisition cost. Recent research shows a very common trend in the development of low-cost cutting force assessment methods for machining processes, due to the high cost of commercial cutting force measurement systems. Some devices that show some promising results in the measurement of these cutting forces have been presented; however, these still require some more testing in order to be as efficient as force sensing systems, such as dynamometers.

Both predictive and measurement methods are employed in the machining process optimization, remaining as a very interesting topic for the industry because it is closely related to productivity performance and quality of the components produced.

There are also two other relevant topics worthy to note: machine learning methods and cutting force assessment in robotic machining. These methods have seen some interesting recent developments and have shown promising results in terms of cutting force assessment:

1. Machine learning models have shown promising results in predicting cutting forces. This approach is quite novel, and it can be seen in the use for cutting force prediction, by using data provided by various sources. These sources can be provided by previous machining tests, such as values from dynamometers readings and simulations. These models need, however, a reliable data source, which can be provided from multiple sources, even from FEM models, enabling the understanding of chip morphology and tool/workpiece temperature, thus providing a better understanding of tool wear. This is very appealing, as it permits the quick optimization of certain machining processes. The more data that are collected, the more reliable the predictions of these methods are.
2. With the increasing use of robots in machining, it makes sense that there is also a need for monitoring cutting forces in these processes, to optimize them. Easy sensor implementation and high versatility of the robot enables process monitoring and correction in real time. However, there is still much room for improvement on this matter, as one of the main drawbacks of this technique is that the robot arm influences the cutting force readings. Indeed, the stiffness of the robot arms influences the cutting force measurements when the robot arm is under severe loads due to the machining process or vibrations of the arm during the operation. The likelihood of process monitoring/optimization in real time, coupled with the high productivity of this process, makes robotic machining an attractive choice over conventional machining; thus, controlling the cutting forces and machining parameters regarding robotic machining is a current focus for the machining manufacturing industry.

## Figures and Tables

**Table 1:** Main research topics in cutting force prediction methods.

Main Research Topics	Advantages/Possibilities	Drawbacks
Improvement of existing methods	-Greater prediction accuracy; -Application of validated method in new machining processes; -Equipment cost is low (when compared to cutting measurement equipment), as it does not require sensors of force sensing systems (if validated).	-Simulation process is time-consuming and may take several hours of computing time; -Harder to implement than cutting force sensing systems (i.e., dynamometers); -Highly reliable on previously collected data.
Creation of new methods and models	-Better understanding of complex machining processes; -Enables the prediction of cutting forces for hard-to-machine materials; -Application on composite machining with reliable results; -Enables the prediction of not only cutting forces, but also other parameters such as tool wear and surface roughness, very important for machining process optimization.	-The employment of simulation methods is very time-consuming, taking several hours of computing time; -Harder to implement than cutting force sensing systems; -Requires validation and is frequently paired with dynamometers for this reason.

**Table 2:** Main research topics of cutting force measurement methods.

Main Research Topics	Advantages/Possibilities	Drawbacks
Employment of dynamometers to study machining processes	-Enables the process optimization; -Deepening of the knowledge of tools’ cutting behavior during machining processes; -Understanding of the behavior of certain materials during machining; -Knowledge on cutting fluid behavior and influence on the overall process.	-High acquisition cost for commercially available dynamometers. -Cutting force obtention is difficult for more complex processes (i.e., five-axis milling or micro-milling); -Influence of equipment and external variables in cutting force readings.
Development of new dynamometers for complex machining processes	-Cutting force obtention for complex machining processes; -Enables for a greater optimization of these processes; -High dynamic behavior (frequencies between 2 and 5 kHz).	-Developed dynamometers require more testing and validation; -Still some interference of external factors, such as vibration and lack of equipment stiffness.
Development of low-cost cutting force measurement systems	More cost-efficient than commercially obtained sensor systems (employment of different sensor configuration and cheaper structure materials); -Ability to optimize a larger number of processes.	-Still need further validation and testing; -Usually dependent on other force-sensing system or simulation for validation; -Low frequency range (inferior to 1 kHz);
Using a sensor system coupled with an analytical/numerical method for cutting force correction	-Obtention of reliable cutting force values; -Improves the dynamic behavior of the system (by introducing filters to correct machining frequencies that interfere with readings); -Can be applied to more complex machining processes, such as five-axis machining; -Enables a greater process optimization, as it can monitor more than just cutting forces.	-High acquisition cost for commercially available dynamometers; -Time-consuming due to the employment of simulations that take several hours of computing time; -Higher complexity, when compared to regular force sensing systems.

**Table 3:** Advantages and drawbacks of recently employed machining process optimization methods.

Process Optimization Method	Advantages	Drawbacks
Experimental methods (machining experiments)	-Accurate value determination; -Process optimization is simpler, requiring only more experiments, if needed.	-Requires multiple experiments, in order to optimize the process; -Low cost-efficiency due to the need for multiple experiments (consumable cost, such as material, tools, and energy consumption); -Optimization takes longer than other methods due to multiple experiments being conducted; -Harder tool monitoring during machining; -Some limitations on some more complex machining processes.
Predictive and numerical method	-Viable value determination; -Lower implementation cost (if validated), as it does not require dynamometers or force sensing systems; -Can be applied to complex machining processes.	-Simulations take several hours of computing time (depending on data); -Usually more complex to implement; -Require accurate previous experimental data; -Require sensor systems for validation, increasing the price of implementation.
Tool-monitoring systems	-Able to monitor tool wear throughout the machining process; -Enables the optimization of tool lifespan; -Enables the development of new tool geometries and coatings; -Can be applied to complex machining processes.	-Systems contain many components. -High implementation cost (when compared to predictive methods), due to the numerous sensors and systems needed to monitor tool wear; -Highly complex implementation due to the number of different sensors and systems used for tool monitoring.
Machine learning models	-Highly flexible, being able to monitor cutting forces and tool wear for multiple machining processes and tools; -Able to continuously learn from simulations and experimentally acquired data; -Accurate predictions.	-Highly reliant on cutting force data; -High cost of development and implementation (when compared to other methods) due to the requirement of experimental data and employment of different types of sensor.

**Table 4:** Common advantages and drawbacks of force-prediction methods.

Advantages	Drawbacks
More cost-efficient when compared to commercially available measuring systems.	Time-consuming, especially in the case of simulations taking several hours of computing time (this being dependent on the quantity of analyzed data).
Able to predict cutting forces in machining of complex parts.	Frequently paired with dynamometers for validation.
Enables machining process optimization.	Highly dependent on collected data.

**Table 5:** Common advantages and drawbacks of force measurement methods.

Advantages	Drawbacks
Reliable cutting force data on simple machining processes.	Some difficulty in cutting force measurement in machining of complex parts.
Enables machining process optimization.	High acquisition cost for commercially available measuring systems (can exceed 100,000 €).
Faster cutting data acquisition when compared to other methods (data is collected during the process, being readily available after it).	Device geometry influences measurement.
